# Effect of tower base painting on willow ptarmigan collision rates with wind turbines

**DOI:** 10.1002/ece3.6307

**Published:** 2020-04-29

**Authors:** Bård G. Stokke, Torgeir Nygård, Ulla Falkdalen, Hans C. Pedersen, Roel May

**Affiliations:** ^1^ Norwegian Institute for Nature Research (NINA) Trondheim Norway; ^2^ Lake Ånnsjön Bird Observatory Duved Sweden

**Keywords:** carcass searches, contrast painting, mitigation measures, mortality, Smøla, willow ptarmigan, wind energy development

## Abstract

1. Birds colliding with turbine rotor blades is a well‐known negative consequence of wind‐power plants. However, there has been far less attention to the risk of birds colliding with the turbine towers, and how to mitigate this risk.
2. Based on data from the Smøla wind‐power plant in Central Norway, it seems highly likely that willow ptarmigan (the only gallinaceous species found on the island) is prone to collide with turbine towers. By employing a BACI‐approach, we tested if painting the lower parts of turbine towers black would reduce the collision risk.
3. Overall, there was a 48% reduction in the number of recorded ptarmigan carcasses per search at painted turbines relative to neighboring control (unpainted) ones, with significant variation both within and between years.
4. Using contrast painting to the turbine towers resulted in significantly reduced number of ptarmigan carcasses found, emphasizing the effectiveness of such a relatively simple mitigation measure.

## Graphical abstract

Willow ptarmigan is prone to collide with turbine towers. Using contrast painting to the turbine towers at the Smøla wind‐power plant in Central Norway resulted in significantly reduced number of ptarmigan carcasses found, emphasizing the effectiveness of such a relatively simple mitigation measure.

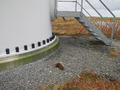

## 1 INTRODUCTION

Renewable energy production is regarded essential to meet the increasing energy demands while also reducing emissions of CO_2_ necessary to reduce risk of global warming (IEA, 2016; IPCC, 2014). In recent years, the production of wind energy has increased worldwide and is still developing fast (IRENA, 2017).

Although regarded as a low‐carbon energy option, wind energy production may cause negative environmental effects, especially on wildlife (Tabassum‐Abbasi, 2014). At onshore wind‐power plants, birds and bats are particularly vulnerable, with effects ranging from mortality caused by collisions with turbines, to displacement/avoidance and habitat loss (Drewitt & Langston, 2006; Langston, Fox, & Drewitt, 2006; May, 2015; Smith & Dwyer, 2016).

Direct mortality of birds due to collision with turbine blades has been reported from many sites (e.g., Loss, Will, & Marra, 2015; de Lucas, Janss, Whitfield, & Ferrer, 2008; de Lucas & Perrow, 2017; Wang, Wang, & Smith, 2015) and may in some species negatively affect population viability (see May, Masden, Bennet, & Perron, 2019). Species particularly vulnerable to collide with turbine blades are those spending time in the air at blade height, such as soaring raptors and birds with aerial displays (e.g., de Lucas & Perrow, 2017). On the other hand, there are few reports of birds colliding with turbine towers.

**FIGURE 1:**
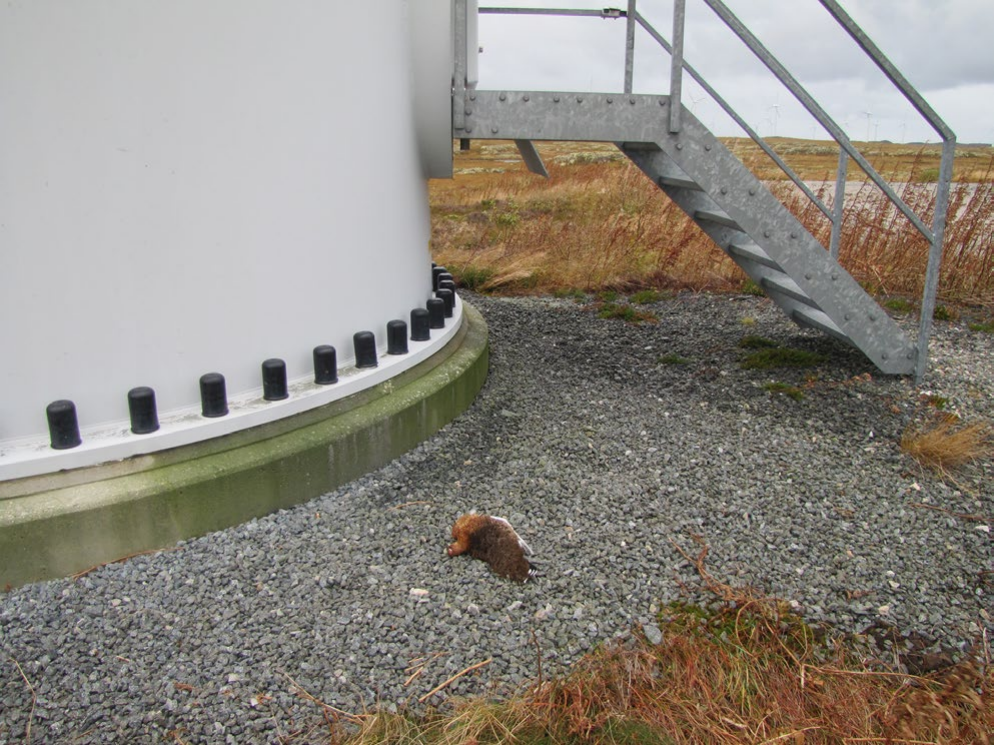
Willow ptarmigan found dead under wind turbine at the Smøla wind‐power plant, Norway in October 2015

Grouse (*Tetraonidae*) are known to have poorly developed vision and flight maneuverability (Rayner, 1988; Sillman, 1973). In addition, many such species are often active during dusk and dawn when visibility is poor. These characteristics all make grouse especially prone to collide with man‐made objects (Bevanger, 1994, 1998; Bevanger & Brøseth, 2000; Bevanger, May, & Stokke, 2016). Studies of grouse in relation to wind‐power plants are addressing both mortality due to collision and displacement due to disturbance (e.g., Hovick, Elmore, Dahlgren, Fuhlendorf, & Engle, 2014; Pruett, Patten, & Wolfe, 2009; Winder, Gregory, McNew, & Sandercock, 2015; Winder et al., 2014a; Winder et al., 2014b; Zeiler & Grunschachner‐Berger, 2009). Considering collisions, carcasses of willow ptarmigan (*Lagopus lagopus*) at the Smøla wind‐power plant are often found only a few meters from the tower base, showing signs of direct impact with a “wall” rather than cuts and fractures usual for hits by turbine blades (Figure 1). In one case, fresh blood smear and feathers was also observed on the tower base where a fresh ptarmigan carcass was found (Bevanger et al., 2010). Galliformes typically fly relatively low above ground; 97% (138 of 142 flights recorded) of willow ptarmigan that were flushed on Smøla, Norway showed a flight height lower than 15 m (Pedersen, 2017). Both data from autopsy and flight height indicate that grouse are more prone to collide with the turbine tower bases than the rotor blades. In support of this, several black grouse (*Tetrao tetrix*) that were found immediately under turbines in an area in Styria, Austria, presumably died because of collision with tower bases and not the rotor blades, even though the cause of death was never observed directly (Zeiler & Grunschachner‐Berger, 2009). Corpses were analyzed by veterinarians, who concluded that injuries were consistent with the birds flying into a hard surface. Furthermore, collision between a willow ptarmigan and the tower base has been confirmed by actual observation once in Sweden (Falkdalen, Lindahl, & Nygård, 2013; Pedersen, 2017), and twice in Scotland (Coppes et al., 2020). In Sweden, it was observed that one individual, part of a group of 10 birds, crashed directly into the tower base at 2.7 m height above ground (25 September 2011, at 07:05 a.m.). The rest of the group passed the tower on both sides. At the time, there was no precipitation or wind, but overcast weather (Falkdalen et al., 2013).

Willow ptarmigan has, due to low population size, been protected from hunting at Smøla from 2005 (Farstad pers. comm.). It is a popular small game species, frequently hunted in Fennoscandia. Due to reduction in population size in most of this species' circumpolar distribution, several restrictions have been introduced on hunting in later years to reduce hunting mortality (Pedersen & Karlsen, 2007; Pedersen & Storaas, 2013; Sandercock, Nilsen, Brøseth, & Pedersen, 2011). The number of planned wind‐power plants is rapidly increasing, not only in coastal areas in Norway, but also in alpine and subalpine areas in Scandinavia. Therefore, any additional negative effects on willow ptarmigan associated with wind turbines are important to assess.

Documenting mortality due to collisions with wind turbines is clearly important, but the next step would be to find solutions to reduce the risk of collisions. It is pivotal to understand why and how birds are killed in order to adopt proper mitigation measures (de Lucas, Ferrer, Bechard, & Muñoz, 2012; de Lucas, Ferrer, & Janss, 2012; Martin, 2011; Wang et al., 2015), and then use this information to select the most efficient tools to reduce bird mortality (Dai, Bergot, Liang, Xiang, & Huang, 2015; Marques et al., 2014; May, Reitan, Bevanger, Lorentsen, & Nygård, 2015).

Norway's largest wind‐power plant at the time was constructed in the period 2002–2005 on the northwestern part of Smøla, an island off the coast of central Norway, consisting of 20 2.0 MW and 48 2.3 MW wind turbines distributed within an 18 km^2^ area (Bevanger et al., 2010; Follestad, Flagstad, Nygård, Reitan, & Schulze, 2007; May, Nygård, Dahl, & Bevanger, 2013). After having first documented the effects of wind turbines on birds within the BirdWind research project (2006–2011; Bevanger et al., 2016), the main aim of the INTACT (INnovative Tools to reduce Avian Collisions with wind Turbines) project (2013–2017) was to advance one step and experimentally test various mitigation measures at the Smøla wind‐power plant. One of these measures was painting of tower bases to increase the contrast of the turbine base against the background, thus making them more visible and easier to avoid for low‐flying ptarmigan (May, 2017).

## 2 MATERIALS AND METHODS

### 2.1 Study location

Smøla consists of a large main island together with about 5,500 smaller islands, islets, and skerries and is located off the coast of Møre and Romsdal County, central Norway (63°24′N, 08°00′E). The terrain is flat with the highest point only 64 m above sea level. Habitats are dominated by moors of heather (*Calluna vulgaris*), marshlands and low rocky outcrops (May et al., 2013).

### 2.2 Search regime

Searches for dead birds below turbines started in August 2006. Dogs trained to find carcasses and remains (e.g., feathers) of birds were used, as this has been shown to increase search efficiency (Paula et al., 2011). An area of ca 120 m radius from turbines was searched. The searches were done in a regular pattern of bands ca 30 m apart, in a transverse pattern perpendicular to the wind direction. The dogs marked the position of remains of birds by lying down on the spot and were then rewarded. Altogether four different trained dogs have been used, and the searcher was always the owner of the dog. Simultaneously, the owners searched the area visually. When an object was found, remains were collected, the presumed species, date, turbine number, distance and direction from the turbine was noted, and the GPS position taken.

**FIGURE 2:**
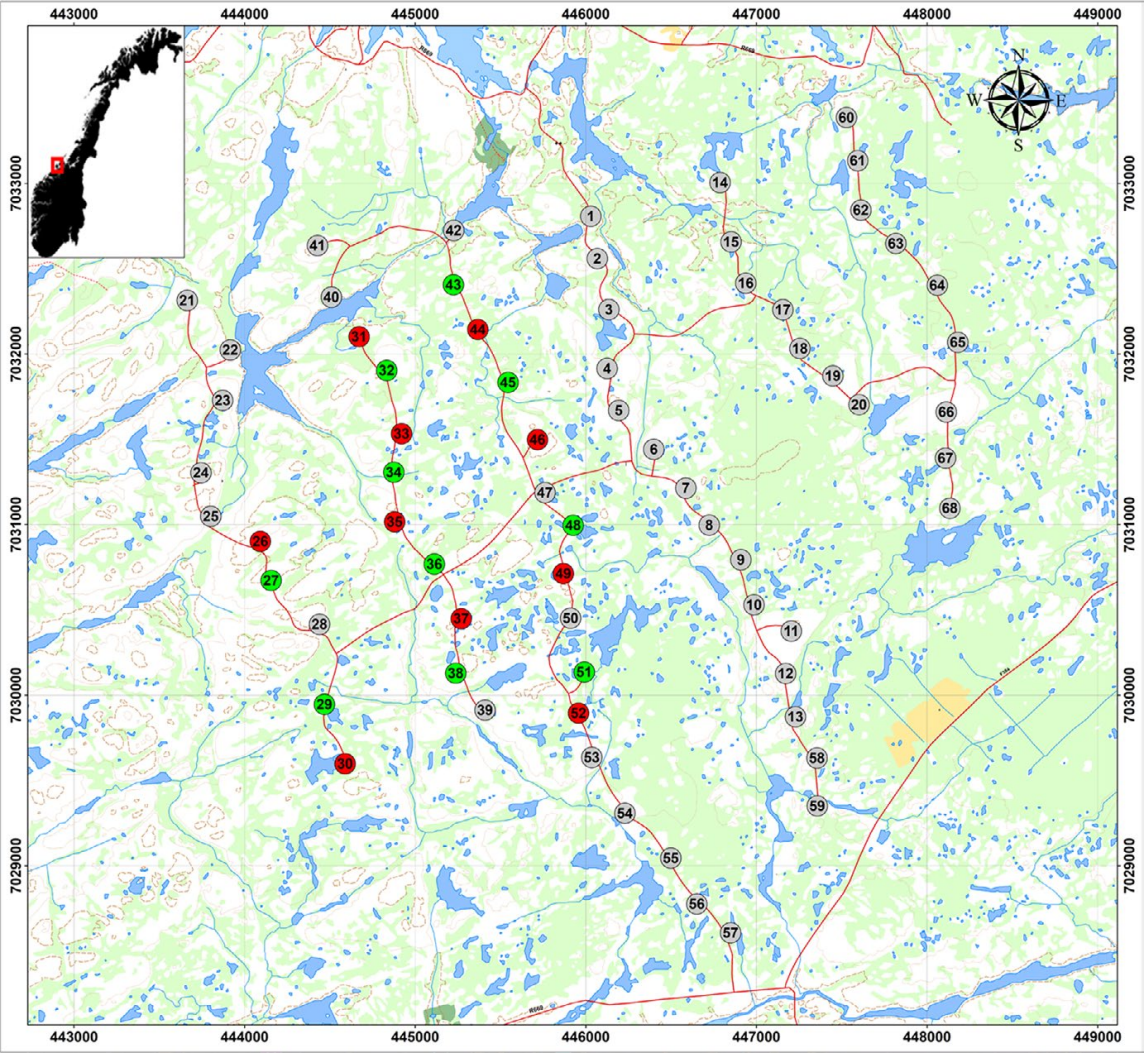
Map of the Smøla wind‐power plant showing position and number of the turbines. Ten turbines had painted tower bases (red) with ten adjacent control turbines (green)

Search intensity has varied over time to address the different research questions of consecutive research projects (BirdWind August 2006—December 2010; searches not connected to projects January 2011—November 2012; INTACT March 2013—March 2017). During August–December 2006, all 68 turbines were searched three out of four weeks every month. In 2007, 25 randomly chosen turbines were searched during 47 weekly searches. During 2008–2012, weekly searches were conducted at the same turbines as in 2007. In 2011, five complete searches were conducted at all 68 turbines (January, April, May, September, and November). In 2012–2013, six complete searches were conducted each year (March, April, May, August, September/October, and November). A new search regime was introduced in the summer of 2014, after experimentally painting black the lower 10 m of the tower base at four turbines in mid‐August 2014. Adjacent turbines were included as control turbines in the searches. Six more turbines had their bases painted black during mid‐July 2015, with neighboring turbines as controls (Figure 2). Searches from the time of painting in 2014 through March 2017 were performed once a month during March–May and August–October. In addition, a full search round (all 68 turbines) was carried out at the end of spring (end of May) and at the end of autumn (end of October). During the winter months, (November–February), a monthly search under all rotor‐swept areas of the turbines was performed. Altogether, 9,424 individual turbine‐searches were performed. The present study only focuses on testing the efficacy of turbines with painted tower bases in reducing collisions of ptarmigan.

### 2.3 Statistical analyses

We tested for any effects of tower base painting on ptarmigan mortality rates (i.e., carcasses found), before and after painting following a Before‐After‐Control‐Impact (BACI) approach. The analyses were performed by grouping recorded number of carcasses per turbine either by year (2006–2017) or by season (winter [December–February], spring [March–May], summer [June–August] and autumn [September–November]). For each turbine, the number of recorded carcasses was calculated as well as the number of searches performed at each turbine. In the analyses, the number of recorded carcasses at ten control turbines (Turbine number: 27, 29, 32, 34, 36, 38, 43, 45, 48, 51) was compared with the painted turbines (Turbine number: 26, 30, 31, 33, 35, 37, 44, 46, 49, 52) before and after painting, while taking into account search effort by including an offset term. To control for any potential effects of turbines and either year or season, random effects were included in a generalized linear mixed‐effects model using the glmer function of the lme4 library with a Poisson distribution (Bates, Machler, Bolker, & Walker, 2015) in the statistical software program R 3.3.2 (R Core Team, 2016). To control for potential overdispersion in the data, we also included an observation‐level random effect (Harrison, 2014). The distribution of turbine distances of ptarmigan carcasses across time and treatment was tested with the Levene's test for homogeneity, using the leveneTest function in the car library (Fox & Weisberg, 2019). Using a similar model structure as for the mortality rates, effects of painting on the average (log‐transformed) distance where ptarmigan carcasses were found were tested using linear mixed‐effects using the lmer function of the lme4 library.

## 3 RESULTS

**FIGURE 3:**
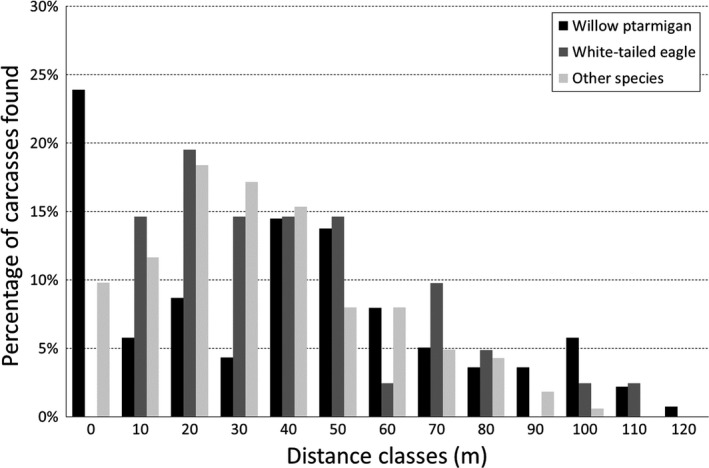
Proportional distribution of recorded carcasses at the Smøla wind‐power plant (2006–2017) by distance from turbine

Altogether, in the period 2006–2017, 474 carcasses were found within the wind‐power plant, including feather heaps or fragmented remains of birds. The dominating species found was willow ptarmigan (*N* = 194), followed by white‐tailed eagle (*Haliaeetus albicilla*) (*N* = 73). Data on distance to turbine was collected for 342 carcasses, 138 willow ptarmigan, 41 white‐tailed eagles, and 163 other species combined (Figure 3).

When regressing distance (log‐transformed, maximum cutoff distance of 120 m) against species using linear regression, and considering all turbines for the whole study period, there were significant species‐specific differences at what distance from the turbine base carcasses were found (*F* = 3.111, *p* = .046). Ptarmigan were found significantly closer to turbines compared to eagles (*t* = −2.265, *p* = .024). The variation among species falling within the grouping “other species” (other than eagles or ptarmigan) led to a nonsignificant effect (*t* = −1.413, *p* = .16). For ptarmigan, 23.9% of all carcasses were found within 10 m of the turbine tower, against 0.0% and 9.8% for white‐tailed eagles and other species, respectively (Figure 3).

When considering the 10 control turbines, there were 11 ptarmigan carcasses found in the period before painting (1,400 searches) and 19 carcasses found in the period after painting (505 searches). Same numbers for the 10 painted turbines were 25 carcasses in the period before painting (1,023 searches) and 14 carcasses in the period after painting (523 searches).

**FIGURE 4:**
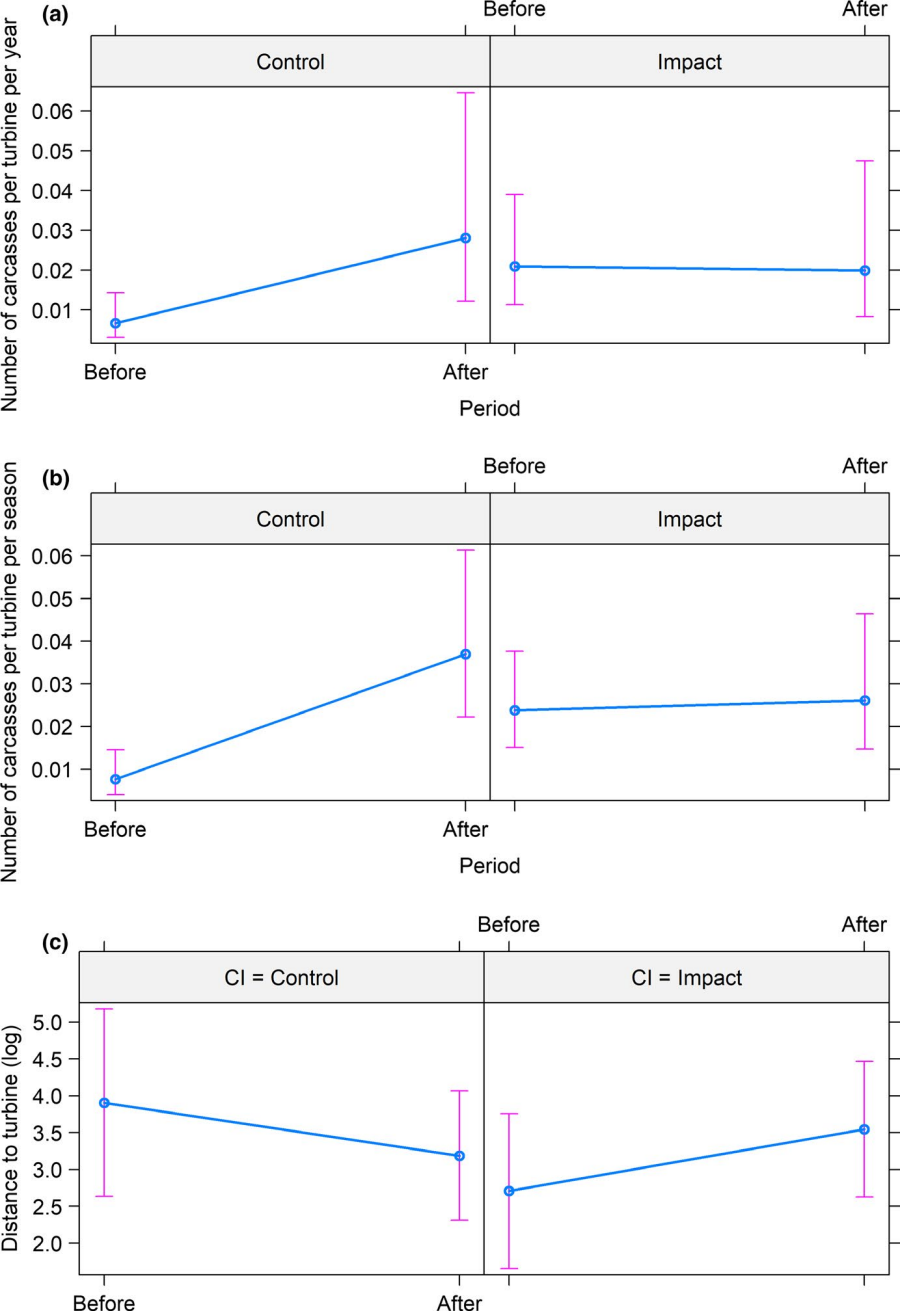
Effect plots testing the effect of painting on the yearly (a) and seasonal (b) rate of ptarmigan carcasses found at the Smøla wind‐power plant using a Before‐After‐Control‐Impact (BACI) design. Panel (c) provides the effect of painting on distances from turbines where carcasses were found. Control = unpainted turbine towers, Impact = painted turbine towers, Before = period before painting, After = period after painting. Estimates are controlled for search effort using an offset term

**FIGURE 5:**
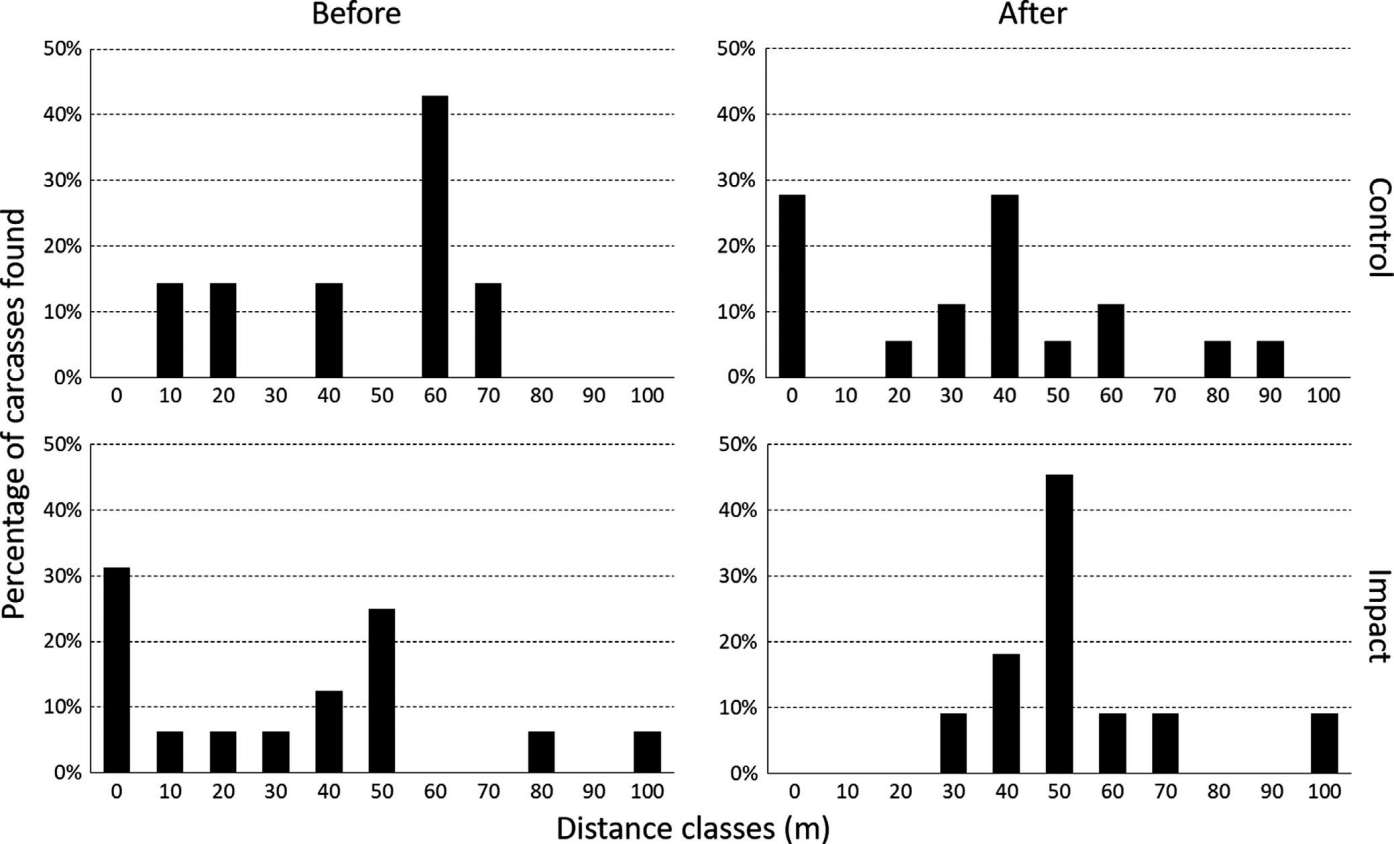
Proportional distribution of willow ptarmigan carcasses found at varying distances from the turbine base at the experimental turbines (10 control and 10 impact turbines) before and after painting within the Smøla wind‐power plant

**FIGURE 6:**
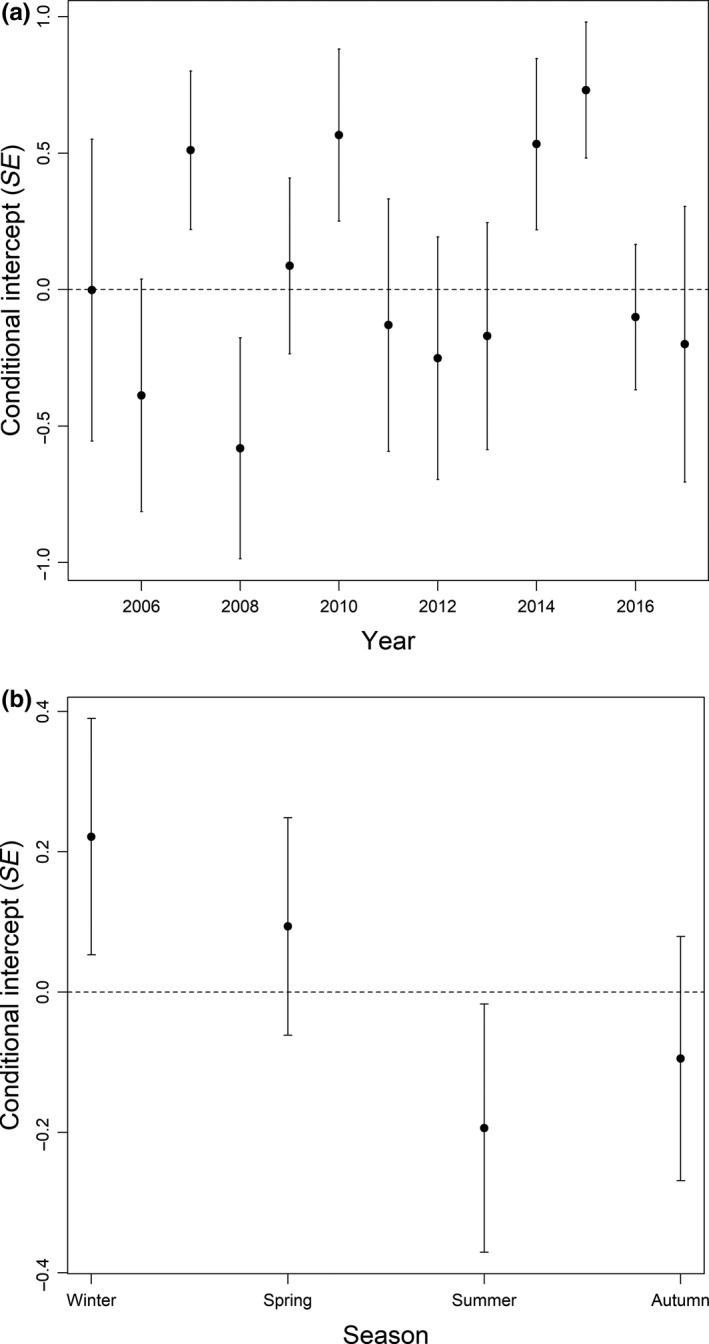
Yearly (a) and seasonal (b) variation in number of ptarmigan carcasses found per search per turbine at the Smøla wind‐power plant as derived from the conditional random intercepts of the linear mixed‐effects model explaining effects of painting of tower bases. Above and below zero indicates, respectively, more or less carcasses found compared to the overall mean. Estimates are controlled for search effort using an offset term

The generalized linear model disclosed that the yearly number of carcasses (across seasons) after painting was reduced at the painted turbines (*z* = −2.884, *p* = .004, Table 1). Overall, there was a 48.2% (95% confidence interval: 44.2%–52.0%) reduction in the annual number of recorded carcasses per search at painted turbines relative to unpainted ones. This effect was mainly due to the relatively large increase in fatalities per search at the control turbines (before: 0.005, after: 0.030), but lack of such a large increase at painted turbines (before: 0.019, after: 0.023) (Figure 4a). We found no effect of birds having a higher probability of collision at the neighboring control turbines due to painting. This was tested by comparing control turbines to other untreated turbines before‐after “treatment” within the wind‐power plant (*z* = 0.283, *p* = .78). The average distance of ptarmigan carcasses from the turbine base increased significantly at the painted turbines after painting (*F* = 6.535, *p* = .014). After painting, no ptarmigan carcasses were found within 30 m of the painted turbines (Figure 5). However, the number of carcasses fluctuated considerably between years (Figure 6a).

**TABLE 1:** Model estimates testing the effect of painting on the yearly (upper table) and seasonal (middle table) rate of ptarmigan carcasses found at the Smøla wind‐power plant using a Before‐After‐Control‐Impact (BACI) design. The lower table provides the effect of painting on distances from turbines where carcasses were found. The models controlled for search effort using an offset term

YEAR				
Fixed effects	Estimate	SE	*z* value	*p*
Intercept	−5.006	0.385	−12.997	<.001
BA—After	1.434	0.512	2.800	.005
CI—Impact	1.142	0.370	3.082	.002
BA:CI	−1.485	0.519	−2.863	.004

Random intercepts	Variance	*SD*	*N*	
Record	0.078	0.279	261	
Turbine	2.695 × 10^−9^	5.191 × 10^−5^	20	
Year	0.306	0.554	13	

SEASON				
Fixed effects	Estimate	SE	*z* value	*p*
Intercept	−4.868	0.324	−15.023	<.001
BA—After	1.569	0.378	4.155	<.001
CI—Impact	1.129	0.361	3.130	.002
BA:CI	−1.475	0.503	−2.931	.003

Random intercepts	Variance	*SD*	*N*	
Record	1.059 × 10^−9^	3.254 × 10^−5^	160	
Turbine	2.476 × 10^−10^	1.573 × 10^−5^	20	
Season	0.054	0.233	4	

DISTANCE (log‐transformed)				
Fixed effects	Estimate	SE	*t* value	*p*
Intercept	3.904	0.634	6.156	<.001
BA—After	−0.718	0.678	−1.060	.301
CI—Impact	−1.197	0.785	−1.526	.136
BA:CI	1.556	0.778	1.999	.051

Random intercepts	Variance	*SD*	*N*	
Turbine	0.747	0.864	18	
Year	0.158	0.397	8	
Season	2.251 × 10^−7^	4.744 × 10^−4^	4	

The seasonal number carcasses (across years) were significantly reduced at the painted turbines (*z* = −3.052, *p* = .002, Table 1), with an overall reduction of 49.0% (95% confidence interval: 44.2%–53.2%). Also here, this seasonal effect was mainly due to the relatively large increase in fatalities per search at the control turbines (before: 0.007, after: 0.037), but lack of such a large increase at painted turbines (before: 0.023, after: 0.027) (Figure 4b). The number of recorded carcasses was higher during winter and spring (Figure 6b). Seasonally, the relative reduction due to painting varied considerably (winter: −31.1% [i.e., increased number of carcasses], spring: 82.9%, summer: 51.5%, autumn: 81.3%).

Testing the distribution of distances of ptarmigan carcasses across time and treatment, there was no significant difference in variances between the groups (*F* = 0.765, *p* = .52). For all bird species taken together, a significant difference in variances was only found when grouping by species (*F* = 11.462, *p* < .001). While the average distance of carcasses at control turbines decreased by 51% (49.6–24.2 m), the average distance at painted turbines increased by 31% (15.0–34.6 m) (Figure 5). Relative to control turbines, carcasses at impact turbines after painting were on average 10.4 m farther away from the turbine base (*t* = 1.999, *p* = .051; Table 1, Figure 4c).

## 4 DISCUSSION

Carcasses of willow ptarmigan were the most frequently observed of all species close to wind turbines at the Smøla wind‐power plant. Ptarmigan carcasses were usually found closer to the turbines relative to other species. Together with the general findings that (a) these carcasses often had injuries that comply with impact from hitting the tower base instead of the turbine blades (Bevanger et al., 2010; Pedersen, 2017), (b) ptarmigan carcasses were found closer to turbines than other species (Figure 3), (c) number of carcasses found close to turbines were lower after painting than before (Figure 3), and (d) overall number of carcasses found declined after painting of the tower bases, strongly indicates that turbine tower base collisions are an important cause of death for this species at the wind‐power plant on Smøla. However, importantly, it is also probable that some ptarmigan collide with the turbine blades, as there is also a peak in the distribution of distance from turbine of carcass at 40–60 m (Figure 3). In addition, predation by raptors such as golden eagles (*Aquila chrysaetos*) and gyrfalcons (*Falco rusticolus*) is an important source of mortality among ptarmigan on Smøla, especially during winter (Bevanger et al., 2010; Brøseth, Nilsen, & Pedersen, 2012). Hence, predation was likely the source of mortality for an unknown proportion of the ptarmigan carcasses found. In a telemetry study on ptarmigan at the Smøla wind‐power plant (Bevanger et al., 2010), collisions with wind turbines accounted for 35.7% of the mortalities, while predation accounted for 42.9%–57.1% (*N* = 28). However, there is no reason to assume that there should be difference in predation pressure at painted versus unpainted turbine towers.

Our results also show that there was a variation in collision risk both among years and seasons (Ferrer et al., 2012; Martin, Perez‐Bacalu, Onrubia, Lucas, & Ferrer, 2018), which has also been found in studies on other bird species colliding with man‐made objects (e.g., Avery, Springer, & Dailey, 1978; Barrios & Rodriguez, 2004; Bevanger & Brøseth, 2000). Annual variation in collision risk in ptarmigan may be influenced by environmental factors such as variation in weather conditions. Spells of weather regimes resulting in fog and rain, for instance, may result in poor visibility and hence a greater danger of colliding with stationary objects (Bevanger, 1994; Drewitt & Langston, 2006). Potentially, fluctuating population sizes could also lead to variation in collision risk, as it has previously been found that the ptarmigan population size on Smøla may vary significantly from year to year (Bevanger et al., 2010). A higher winter mortality can cause reduction in number of breeding pairs and hence chick production and vice versa (Pedersen, 1984). Also, the chick production can vary greatly from year to year due to weather conditions and other environmental factors (Kvasnes, Pedersen, Storaas, & Nilsen, 2014). In years with larger ptarmigan population sizes, the probability of finding birds colliding with turbines could increase simply due to more individuals using the air space in the area. However, there is no indication that the population size on Smøla has declined after the construction of the wind‐power plant. Furthermore, both the density of ptarmigan and chick production inside the power plant has not been significantly different from outside (Bevanger et al., 2010).

Seasonal variation in collision risk may also be influenced by weather since amount and nature (i.e., rain or snow) of precipitation, wind speed, etc. may be connected to season. In addition, variation in light conditions and species‐specific behavioral patterns may explain seasonal variation in collision risk (e.g., Bevanger, 1994). Bevanger (1995) found a peak of collisions with power lines for black grouse in the autumn (September to October), while most collisions in willow ptarmigan occurred in winter and early spring (November to March). This fits well with the results from the Smøla wind‐power plant, where collisions most frequently occurred in winter and spring (Figure 5). This is also supported by earlier findings of the same willow ptarmigan population by Brøseth et al. (2012).

The effect of painting of the turbine tower bases was most pronounced in spring and autumn. The lack of effect of painting during winter could be due to generally poor light conditions, making tower bases hard to observe no matter their appearance (Pedersen, 2017). At 63°N, the daylight hours in the period November to March spans 4.5–10 hr, while at maximum in June it spans 20.5 hr (https://www.timeanddate.no/astronomi/sol/). Elevated collision risk during autumn and spring compared to summer could be due to generally higher flight activity during the former periods. During spring, much territorial behavior includes frequent flying during dusk and dawn, in autumn brood break up, flocking and movements between areas, whereas in the summer time ptarmigan spend most of their time on ground (Pedersen & Karlsen, 2007).

Failure to detect carcasses may obviously influence estimation of collision frequencies. At the control turbines, there was an increase in the number of carcasses found after painting than before (Figure 4). This result is difficult to explain, but it could be due to variation in search efficiency. In the present study, the search regime was constant throughout the whole search period, and variation in search intensity was controlled for in the analyses. In addition, searches were only made by trained dogs and in accordance with a standardized protocol. One person with a trained dog searched for carcasses before painting, and another person with another dog carried out the search after painting. However, if the post painting search team was more efficient in finding carcasses, this should only lead to an underestimation of the effect of painting and hence the results are even stronger in favor of the positive effect of painting.

Another possible reason for the increase in number of carcasses found at the control turbines before and after painting could be that ptarmigan showed anticipatory evasion (cf. May et al., 2015) of the painted turbines by changing flight paths, and were therefore more likely to collide with the unpainted turbines. However, we found no indications of ptarmigan being “forced into” neighboring turbines due to an evasive response to the painted rotor blades. This possibility, however, merits further investigations focusing on ptarmigan movements within the study area.

Scavenger removal of collision victims may obviously influence estimation of collision frequencies (e.g., Loss et al., 2015). The introduced American mink (*Neovison vison*) is the only mammalian scavenger observed within the Smøla wind‐power plant. There are no foxes (*Vulpes* spp.) or weasels (*Mustela* spp.) on the island. Therefore, the only factors affecting the carcasses are scavenging birds (corvids), mink, and insects (maggots) (Bevanger et al., 2010). Carcass removal experiments were carried out on Smøla in November 2010. Twenty‐three ptarmigan carcasses were fitted with radio‐transmitters and a camera‐trap was put up at all carcasses. Results showed that 26.1% of ptarmigan carcasses were removed within the first two weeks after initiation of the experiment (Bevanger et al., 2010). As for search bias, there is no reason to believe that scavenger removal rates should vary significantly between years or between painted and control turbines.

In summary, the present study represents the first case documenting that painting of the wind turbine tower base reduces bird collisions. This relatively simple and cost‐effective mitigation measure should be considered in the planning of new wind‐power plants, especially in areas where Galliformes and other birds with relatively poor vision and maneuverability, and that generally perform low altitude flights are occurring.

## CONFLICT OF INTEREST

The authors declare no conflict of interest.

## AUTHOR CONTRIBUTION

**Bård Gunnar Stokke:** Investigation (equal); Supervision (equal); Writing‐original draft (lead); Writing‐review & editing (lead). **Torgeir Nygård:** Conceptualization (equal); Data curation (equal); Formal analysis (equal); Investigation (equal); Methodology (equal); Supervision (equal); Writing‐original draft (supporting); Writing‐review & editing (supporting). **Ulla Falkdalen:** Investigation (equal); Methodology (equal); Writing‐original draft (supporting); Writing‐review & editing (supporting). **Hans Christian Pedersen:** Conceptualization (equal); Investigation (equal); Methodology (equal); Supervision (equal); Validation (equal); Writing‐original draft (supporting); Writing‐review & editing (supporting). **Roel May:** Conceptualization (lead); Data curation (equal); Formal analysis (lead); Funding acquisition (lead); Investigation (lead); Methodology (lead); Project administration (lead); Supervision (equal); Validation (lead); Visualization (lead); Writing‐original draft (supporting); Writing‐review & editing (supporting).

## Data Availability

The data that support the findings of this study have been deposited in Dryad with https://doi.org/10.5061/dryad.7wm37pvq1.
